# Regulation of IL-6 Secretion by Astrocytes via TLR4 in the Fragile X Mouse Model

**DOI:** 10.3389/fnmol.2018.00272

**Published:** 2018-08-03

**Authors:** Victoria Krasovska, Laurie C. Doering

**Affiliations:** Department of Pathology and Molecular Medicine, McMaster University, Hamilton, ON, Canada

**Keywords:** astrocyte, development, fragile X syndrome, Tenascin C, TLR4, IL-6, synapses

## Abstract

Fragile X syndrome (FXS) is identified by abnormal dendrite morphology and altered synaptic protein expression. Astrocyte secreted factors such as Tenascin C (TNC), may contribute to the synaptic changes, including maturation of the synapse. TNC is a known endogenous ligand of toll-like receptor 4 (TLR4) that has been shown to induce the expression of pro-inflammatory cytokines such as interleukin-6 (IL-6). At the molecular level, elevated IL-6 promotes excitatory synapse formation and increases dendrite spine length. With these molecular changes linked to the phenotype of FXS, we examined the expression and the mechanism of the endogenous TLR4 activator TNC, and its downstream target IL-6 in astrocytes from the Fragile X Mental Retardation 1 (*FMR1*) knockout (KO) mouse model. Secreted TNC and IL-6 were significantly increased in *FMR1* KO astrocytes. Addition of TNC and lipopolysaccharide (LPS) induced IL-6 secretion, whereas the antagonist of TLR4 (LPS-RS) had an opposing effect. Cortical protein expression of TNC and IL-6 were also significantly elevated in the postnatal *FMR1* KO mouse. In addition, there was an increase in the number of vesicular glutamate transporter 1 (VGLUT1)/post synaptic density protein 95 (PSD95) positive synaptic puncta of both wild-type (WT) and *FMR1* KO neurons when plated with astrocyte conditioned media (ACM) from *FMR1* KO astrocytes, compared to those plated with media from wild type astrocytes. By assessing the cellular mechanisms involved, a novel therapeutic option could be made available to target abnormalities of synaptic function seen in FXS.

## Introduction

Fragile X Syndrome (FXS) is the most common inherited form of autism which affects 1:4,000 males and 1:8,000 females (Hatton et al., 1998; Jin and Warren, 2000; Duy and Budimirovic, 2017). A multitude of symptoms occur in FXS ranging from mild to severe behavioral deficiencies such as cognitive impairment, hyperactivity, anxiety, autistic-like behaviors, susceptibility to seizures and motor disorders (Contractor et al., 2015; Hunsaker, 2012). The defects in individuals with FXS are attributed to the mutation in the Fragile X Mental Retardation 1 (*FMR1*) gene resulting from the expansion of the CGG untranslated region of the X chromosome (Jin and Warren, 2000). This expansion leads to the hypermethylation and silencing of the *FMR1* gene, preventing the synthesis of the FMRP (Jin and Warren, 2000).

FMRP is an mRNA binding protein which regulates the translation, and transport of mRNA in the brain, with the highest levels seen in hippocampal and cerebellar neurons (Pacey et al., 2015). The lack of FMRP in FXS has been associated with abnormal synapse formation, synapse number and structure (Pfeiffer and Huber, 2009; Yang et al., 2016). In neurons, FMRP is known to regulate synaptic plasticity; in particular metabotropic glutamate receptor (mGluR) mediated long-term depression (LTD; Contractor et al., 2015). Under normal conditions FMRP is expressed in neurons (Arsenault et al., 2016), astrocytes and oligodendrocytes where it influences the synaptic environment to bind, transport and translocate mRNA (Wang et al., 2004).

Recently, astrocytes have emerged as an important cell type for regulating the synaptic environment, neuronal activation and plasticity through astrocyte secreted factors (Jones and Bouvier, 2014). Tenascin C (TNC) is an extracellular matrix glycoprotein secreted by astrocytes and has been implicated in extracellular matrix re-modeling during tissue repair and synapse development (Jones and Bouvier, 2014; Stamenkovic et al., 2016). Non-neuronal cells stimulated with exogenous TNC exhibited increased levels of pro-inflammatory cytokines such as tumor necrosis factor alpha (TNF alpha), interleukin-6 (IL-6) and IL-8, via Toll-like receptor 4 (TLR4) activation (Midwood et al., 2009; Maqbool et al., 2016). TNC has also been investigated in blood-brain barrier (BBB) repair (Fujimoto et al., 2016) and regulation of inflammatory cytokines (Latijnhouwers et al., 1998). In support of this, TLR4 is expressed at the surface of astrocytes and microglia, and has been found to induce inflammation in neurological diseases (Trotta et al., 2014).

Preliminary results suggest that TLR4 stimulation in astrocytes, increases the secretion of the pro-inflammatory cytokine IL-6 (El-Hage et al., 2011). Elevated levels of IL-6 have been implicated in cognitive impairments, behavioral deficits and decreased social interactions (Greenhill et al., 2011). At the molecular level, elevated IL-6 increases excitatory synapse formation, while impairing the development of inhibitory synapses (Wei et al., 2013; Shen et al., 2016). In addition, IL-6 has been shown to increase the length of dendritic spines and stimulate the formation of mushroom shaped dendritic spines, implying that IL-6 is involved in synapse formation (Wei et al., 2012).

Recent data suggest the action of three distinct regions of the C-terminal fibrinogen like globe (FBG) domain of TNC act cooperatively to activate TLR4, thereby promoting cytokine synthesis (Zuliani-Alvarez et al., 2017). Given the respective role of TLR4 activation in non-neuronal tissue, the same principle may be applied to TLR4 expressed by astrocytes. TLR4 activation may influence synaptic development, therefore resulting in abnormal formation and maturation of excitatory synapses in FXS. Here we compared the developmental cortical expression of TNC, TLR4 and IL-6 from postnatal day (P), ranging from P1 to P21 in wild-type (WT) mice and mice that lack FMRP (*FMR1* knockout, KO). In addition, astrocytes were treated with exogenous TNC and Lipopolysaccharide (LPS) to determine the function of TLR4 activation in astrocytes. Treatments of cultured astrocytes with known antagonist of TLR4, LPS from *Rhodobacter sphaeroides* (LPS-RS) were conducted to determine if inflammatory cytokine synthesis could be decreased with the addition of an antagonist. Lastly, the role of astrocyte conditioned media (ACM) on synaptic development of WT and *FMR1* KO neurons was investigated. Most importantly, our findings suggest the expression of TNC and IL-6 are dysregulated in the cortical regions in FXS. With TNC contributing to induction of IL-6 through the activation of astrocytic TLR4, this mechanism may contribute to abnormal synapse development.

## Materials and Methods

### Animals

WT and *FMR1* KO mice (FVB.129P2[B6]-Fmr1 tm1Cgr) were housed and bred in the McMaster University Central Animal Facility. All experiments and animal-handling procedures followed the guidelines set by the Canadian Council on Animal Care and were approved by the McMaster Animal Research Ethics Board (AUP 13-12-49).

### Cortical Tissue Isolation for Western Blotting

WT and knockout (*FMR1* KO) mice were decapitated and whole brains were extracted at P1 (six males and two females), P7 (five males and three females), P14 (six males and two females) and P21 (eight males and zero female) into ice cold, sterile phosphate-buffered saline (PBS, 0.01 M). Cortical samples were dissected and were immediately flash-frozen in isopentane and stored at −80°C. Samples were mechanically homogenized on ice in lysis buffer (150 mM NaCl, 1% NP40, 0.5% Deoxycholic Acid, 1% SDS, 50 mM Tris, Roche ULTRA protease inhibitor tablet, Roche PhoSTOP phosphatase inhibitor tablet). Homogenized tissue samples were left on ice for 1 h and centrifuged at 16,000 rpm for 15 min at 4°C. The supernatant was collected and the protein concentration of each sample was determined by a DC protein assay (Bio-Rad, Mississauga, ON, Canada). Samples were aliquoted and stored at −80°C.

### Primary Cortical Astrocyte Culture

Primary cultures of astrocytes were isolated from *FMR1* KO and WT mice as described by Jacobs and Doering (2009). Cortical astrocytes were isolated from WT and *FMR1* KO mice at P1 or P2 and grown in a T75 culture flask in minimum essential media (Invitrogen, Carlsbad, CA, USA), supplemented with 30% glucose and 10% horse serum (Invitrogen). Culture were maintained for approximately 1 week or until 70% confluent at 37°C and 5% CO_2_. Cells were then removed from the T75 flask with the addition of Trypsin-EDTA 0.05% (Invitrogen) and re-plated on 24-well plates with coverslips for immunocytochemistry experiments or onto 6-well plates for collection of ACM. The 24-well plate contained coverslips coated with Poly-L-Lysine (Sigma-Aldrich, St. Louis, MO, USA; 1 mg/mL) and laminin (Invitrogen; 0.1 mg/mL) and seeded with 5,000 cells per well. Cells were maintained for 3 days *in vitro* for subsequent immunocytochemistry. The 6-well plates were also coated with Poly-L-Lysine (Sigma-Aldrich, St. Louis, MO, USA; 1 mg/mL) and laminin (Invitrogen; 0.1 mg/mL) and seeded at a density of 100,000 cells per well. Cells were maintained for 3 to 5 days *in vitro* for analysis of ACM and cell collection via Western Blotting.

### Western Blotting

**Figure 1:**
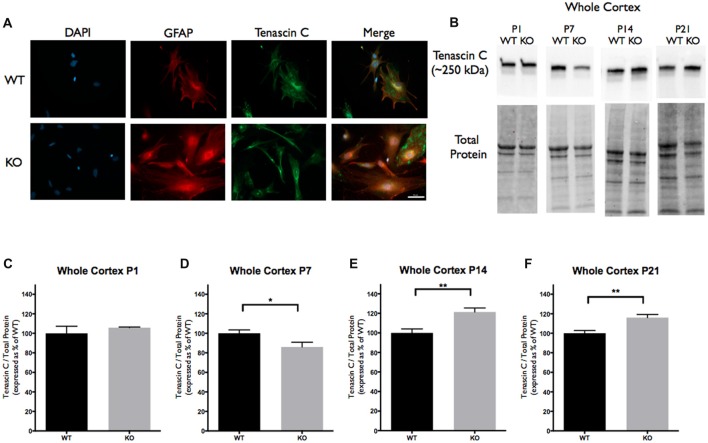
Tenascin C (TNC) expression is significantly altered at postnatal period P7–P21 in the cortex of Fragile X Mental Retardation 1 (*FMR1*) knockout (KO) mice. **(A)** Cultured cortical astrocytes co-labeled with anti-glial fibrillary acidic protein (GFAP; red), 4’,6-diamidino-2-Phenylindole (DAPI; blue) and anti-TNC (green) after 3 days *in vitro*. Images were obtained using a 40× objective with a Zeiss Axioimager M2. Scale bars = 50 μm. **(B)** Western blots showing TNC expression (~250 kDa) in cortical samples containing 30 μg of protein from P1, P7, P14 and P21 wild-type (WT) and *FMR1* KO mice. Corresponding total protein was used as a loading control. **(C–F)** Cortical expression of TNC in WT (black; *n* = 8) and *FMR1* KO (gray; *n* = 8) mice at P1 (six males and two females), P7 (five males and three females), P14 (six males and two females) and P21 (eight males and zero female). Representative bands were normalized to the total protein and expressed as a percentage of the average TNC in the WT group. Statistical differences denoted with a single asterisk, *P* < 0.05 and with a double asterisk, *P* < 0.01.

**Figure 2:**
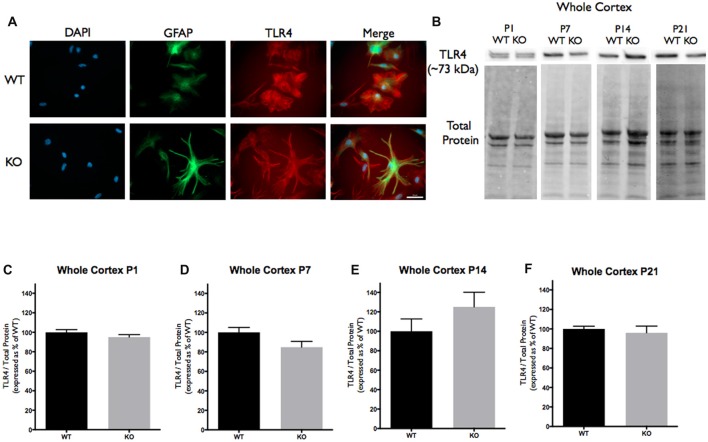
Toll-like receptor 4 (TLR4) expression is not significantly altered in the cortex of *FMR1* KO mice. **(A)** Cultured cortical astrocytes co-labeled with anti-GFAP (green), DAPI (blue) and anti-TLR4 (red) after 3 days *in vitro*. Images were obtained using a 40× objective with a Zeiss Axioimager M2. Scale bars = 50 μm. **(B)** Western blots showing TLR4 expression (~73 kDa) in cortical samples containing 30 μg of protein from P1, P7, P14 and P21 WT and *FMR1* KO mice. Corresponding total protein was used as a loading control. **(C–F)** Cortical expression of TLR4 in WT (black; *n* = 8) and *FMR1* KO (gray; *n* = 8) mice at P1 (six males and two females), P7 (five males and three females), P14 (six males and two females) and P21 (eight males and zero female). Representative bands were normalized to the total protein and expressed as a percentage of the average TLR4 in the WT group.

**Figure 3:**
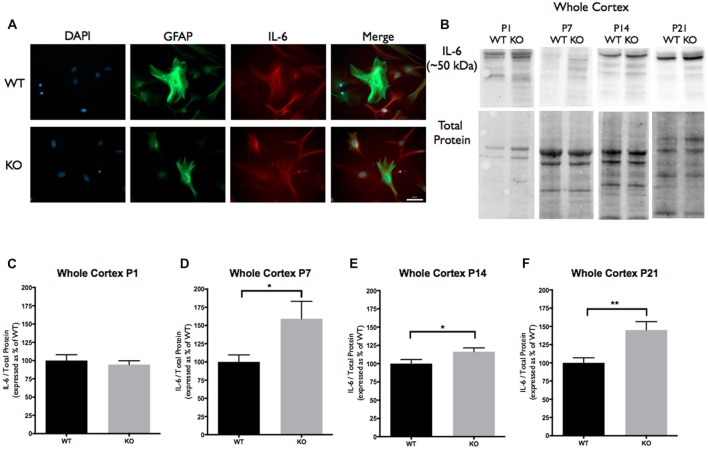
Interleukin-6 (IL-6) expression is significantly increased at postnatal day P7, P14 and P21 in the cortex of *FMR1* KO mice. **(A)** Cultured cortical astrocytes co-labeled with anti-GFAP (green), DAPI (blue) and anti-IL-6 (red) after 3 days *in vitro*. Images were obtained using a 40× objective with a Zeiss Axioimager M2. Scale bars = 50 μm. **(B)** Western blots showing IL-6 expression (~50 kDa) in cortical samples containing 30 μg of protein from P1, P7, P14 and P21 WT and *FMR1* KO mice. Corresponding total protein was used as a loading control. **(C–F)** Cortical expression of IL-6 in WT (black; *n* = 8) and *FMR1* KO (gray; *n* = 8) mice at P1 (six males and two females), P7 (five males and three females), P14 (six males and two females) and P21 (eight males and zero female). Representative bands were normalized to the total protein and expressed as a percentage of the average IL-6 in the WT group. Statistical differences denoted with a single asterisk, *P* < 0.05 and with a double asterisk, *P* < 0.01.

Cortical samples containing 30 μg (homogenized whole tissue) of protein were combined with sample buffer (1×; 5% Beta-Mercaptoethanol+ Laemmli Sample Buffer, Bio-Rad). Samples were boiled at 95°C for 5 min, briefly centrifuged and loaded onto 4%–15% precast polyacrylamide stain-free gel (Bio-Rad). Proteins were separated via electrophoresis at 135V, activated with UV light (302 nm) for visualization of total protein (1 min) and transferred onto polyvinylidene difluoride (PVDF) membrane (Bio-Rad) using the Trans-Blot Turbo Transfer System (Bio-Rad). The membranes were then imaged for total loaded protein using a ChemiDoc Imaging System (Bio-Rad, Mississauga, ON, Canada), after which they were blocked for 1 h in a blocking solution (5% non-fat milk solution in Tris-buffered saline solution with Tween-20 (TBS-T). Each membrane was then incubated overnight at 4°C in TBS-T containing anti-rat TNC antibody (host rabbit; 1:250; MAB2138; R&D Systems), anti-mouse TLR4 antibody (host rabbit; 1:250; 76B357.1; abcam) or anti-mouse IL-6 antibody (host rabbit; 1:500; ab9324; abcam). These antibodies recognize bands at 250 kDa corresponding to TNC (Figure 1B), 73 kDa corresponding to TLR4 (Figure 2B) and 50 kDa corresponding to IL-6 (Figure 3B). Following incubation, the membranes were washed 3 × 10 min in TBS-T and then incubated in TBS-T containing horseradish peroxidase conjugated secondary antibody against rat (1:10,000; ab97057; abcam) or mouse (1:5,000; NA931V; GE Healthcare Life Sciences, Mississauga, ON, Canada) for 1 h at room temperature. Following the incubation period, membranes were then washed 3 × 10 min in TBS-T and developed using enhanced chemiluminescence developer solutions (Bio-Rad). Membranes were scanned using a ChemiDoc Imaging System (Bio-Rad, Mississauga, ON, Canada). Densitometry measurements were conducted using Image Lab Software 5.2 (Bio-Rad). Each band corresponding to either TNC (~250 kDa), TLR4 (~73 kDa), or IL-6 (~50 kDa) was first normalized to total protein within the same lane and then to a cross gel control. These values were then expressed as a relative percentage of the average densitometry value obtained from the age-matched WT samples.

### Cortical Neurons Maintained With Cortical Astrocyte Conditioned Media

Cortical astrocytes were isolated from WT and *FMR1* KO mice at P1 or P2 and grown in a T75 culture flask in minimum essential media (Invitrogen, Carlsbad, CA, USA), supplemented with 30% glucose and 10% horse serum (Invitrogen). Culture were maintained until 70% confluent at 37°C and 5% CO_2_. After confluency has been reached, the media was switched to neural maintenance media (NMM; Stemcell Technologies), and supplemented with SM1 (Stemcell Technologies), GlutaMAX (Gibco) and 2 mg/ml L-Glutamic Acid (Sigma). The media was conditioned for 3 days before it could be used to supplement neuronal cultures. WT and *FMR1* KO neurons were plated on coverslips in a 24-well plate with Poly-L-Lysine (Sigma-Aldrich, St. Louis, MO, USA; 1 mg/mL) and laminin (Invitrogen; 0.1 mg/mL), and seeded at a density of 10,000 cells per well in the conditioned NMM. Neuronal cultures were maintained for 12 days at 37°C and 5% CO_2_ and then processed for immunocytochemical analysis.

### Immunocytochemistry

Immunocytochemistry experiments were carried out with primary cortical astrocyte cultures following a protocol previously described by Cheng et al. (2016). The astrocytes were fixed with 4% PFA for 20 min at room temperature. The cells were washed three times with PBS and permeabilized with 0.1% Triton-X-100. Non-specific binding was blocked with 1.0% Bovine Serum Albumin (BSA) for 30 min at room temperature. Primary antibodies were prepared in PBS and applied to the coverslips for 24 h. The following primary antibodies were used: anti-rat TNC antibody (host rabbit; 1:50; MAB2138; R&D Systems), anti-mouse TLR4 antibody (host rabbit; 1:500; 76B357.1; abcam), anti-mouse IL-6 antibody (host rabbit; 1:1,000; ab9324; abcam) and chicken anti-glial fibrillary acidic protein (anti-GFAP; 1:2,000; CH22102; Neuromics, Minneapolis, MN, USA). The second day, the coverslips were washed three times with PBS, following incubation with the secondary antibody for 3 h at room temperature. These included goat anti-rat Alexa Fluor 488 (1:200; ab150157; abcam), donkey anti-mouse Alexa Fluor 594 (1:1,000; A-21203; Invitrogen), donkey anti-chicken FITC (1:100; 703-095-155; Jackson). Lastly, the coverslips were washed three times with water and PBS, following mounting with ProLong Gold Antifade Mountant with 4’,6-diamidino-2-phenylindole (DAPI; Life Technologies, Carlsbad, CA, USA). Three independent (*n* = 3) cultures and a total of 27 cells were examined per genotype. Visual imaging was acquired using a Zeiss Axioskop 2 epifluorescence microscope and Axiovision (v4.6) image acquisition software.

In addition, neuronal cultures supplemented with conditioned NMM, were processed in a similar manner to determine the co-localized vesicular glutamate transporter 1 (VGLUT1) pre-synaptic and post synaptic density protein 95 (PSD95) post-synaptic puncta. The following primary antibodies were used: guinea pig anti-VGLUT1 (1:200; AB5905; Millipore) and mouse anti-PSD95 (1:100; MAB1596; Millipore). The corresponding secondary antibodies were used: donkey anti-mouse Alexa Fluor 594 (1:1,000; A-21203; Invitrogen) and donkey anti-guinea pig FITC (1:200, 706-095-148, Jackson ImmunoResearch). Sixteen independent neuronal cultures (*n* = 4/condition) supplemented with conditioned NMM were examined. Ten neurons per condition were examined from 9 wells per *n*, resulting in synapse counts averaged from 40 neurons.

### Measurements of Tenascin C and IL-6 Expression and Secretion

TNC and IL-6 levels were determined in astrocyte cultures and in ACM. Confluent astrocyte cultures were incubated with 0.05% Trypsin-EDTA for 5 min at 37°C. Once cells were fully lifted, MEM media supplemented with serum was added to prevent further digestion. The samples were then centrifuged at 250× *g* for 5 min at RT. The cells were then lysed with lysis buffer (150 mM NaCl, 1% NP40, 0.5% Deoxycholic Acid, 1% SDS, 50 mM Tris, Roche ULTRA protease inhibitor tablet, Roche PhoSTOP phosphatase inhibitor tablet). The lysates were collected in Eppendorf tubes and centrifuged at 15,000 rpm for 15 min at 4°C. The supernatant was stored at −80°C until further protein quantification to determine the cell associated concentrations.

Aliquots of ACM from the same culture were processed to determine the secreted (extracellular) TNC and IL-6 levels. In preparation for conditioning, ACM was harvested for 3 days with the MEM media replaced with serum free minimum-essential media on the third day, after which the sample was collected and concentrated. The media was filtered using a 0.22 μm syringe filter for the removal of cellular debris and concentrated using Vivaspin 20 (GE Healthcare) through a 50 kDa molecular weight cut-off (MWCO) for TNC or Vivaspin 6 (GE Healthcare) through a 10 kDa MWCO for IL-6. ACM was stored at −80°C until further protein quantification with Western Blotting.

### Cortical Astrocyte Drug Treatments

To assess TLR4 activation, WT and *FMR1* KO cortical astrocyte cultures were treated independently with exogenous TNC, in addition to the known TLR4 agonist (LPS) to test IL-6 induction. Additionally, TLR4 antagonist treatments with LPS-RS were performed to examine for a decrease cytokine expression. The astrocytes were grown for 3 to 5 days *in vitro* in serum-free minimum essential media (Invitrogen, Carlsbad, CA, USA) supplemented with 30% glucose, achieving 70% confluency. At 3, 6 and 24 h prior to cell and ACM collections, astrocytes were treated with exogenous chicken TNC (10 μg/mL; CC118, Millipore), LPS (10 μg/mL; Sigma-Aldrich) and LPS-RS (10 μg/mL; Sigma-Aldrich), which were added to the astrocyte culture media. The cells were trypsinized and collected as previously discussed. The ACM was filtered using a 0.22 μm syringe and concentrated through a 10 kDa MWCO using Vivaspin 6 (GE Healthcare). The cell associated and secreted IL-6 protein levels were quantified using Western Blotting.

### Synaptic Puncta Analysis

Visual imaging was acquired using a Zeiss Axioskop 2 epifluorescence microscope and Axiovision (v4.6) image acquisition software. SynapCountJ, is a custom written plug-in for ImageJ (National Institutes of Health, Bethesda, MD, USA) used to identify co-localized puncta. Background was removed from both the red and green channels of each image using the ImageJ rolling ball background subtraction algorithm. Dendrite tracings were done using NeuronJ (an ImageJ plugin). The coordinates of these tracings were uploaded into SynapCountJ along with the corresponding red and green channel images. The number of co-localized puncta was measured for each tracing and normalized to the tracing length. The investigators were blind to the genotypes and treatments when investigating the synaptic phenotypes in cultures.

### Statistical Analysis

Statistical analysis was conducted using GraphPad Prism Software 7.0 (GraphPad Software Inc., San Diego, CA, USA). Unpaired, two-tailed *t*-tests were used to identify the differential expression of TNC, TLR4 and IL-6 between the WT and *FMR1* KO groups. Additionally, unpaired, two-tailed *t*-tests were used to identify differences of cell associated and secreted TNC and IL-6 expression of cultured cells and ACM. To determine statistical significance of the treatment groups and synaptic analysis, a two-way ANOVA, along with Tukey’s multiple comparison tests were conducted. All results except for synaptic analysis are shown as mean ± SEM. Synaptic analysis are shown as a box and whiskers plot representing the mean with the interquartile range, with error bars representing the min and max values. Results deemed significant when probability values *P* < 0.05.

## Results

In this study we investigated the developmental expression of TNC, TLR4 and IL-6 in cortical brain regions of WT and *FMR1* KO mice, aged P1, P7, P14 and P21. We hypothesized that the levels of astrocyte derived TNC are dysregulated in the *FMR1* KO mouse model, thus contributing to the elevated IL-6 levels, which may be responsible for the aberrant synaptic changes seen in FXS. While the expression of TLR4 remained consistent between the genotypes, treatments with LPS and exogenous TNC suggest that TLR4 is responsible for IL-6 secretion in astrocytes.

### Tenascin C and IL-6 Protein Levels Are Altered in the Cortex of *FMR1* KO Mice

TNC, TLR4 and IL-6 were all highly expressed in primary cortical astrocytes from the WT and *FMR1* KO P1 or P2 mice. All three targets showed similar distribution patterns between the groups following 3 days *in vitro* (*n* = 3, 27 cells/group; Figures 1A, 2A, 3A). To determine if there was differential expression of TNC, TLR4 and IL-6 in *FMR1* KO mice at P1, P7, P14 and P21, Western blotting experiments were conducted. There was a decrease of TNC expression between the WT and *FMR1* KO groups at P7 (86.03 ± 4.827, *P* < 0.05; *n* = 8/group; Figures 1B,D). At P14, TNC levels were increased in the *FMR1* KO group (121.4 ± 4.087, *P* < 0.01; *n* = 8/group; Figures 1B,E). Additionally, TNC levels were elevated in the *FMR1* KO group at P21 (116.1 ± 3.179, *n* = 8; *P* < 0.01; *n* = 8/group; Figures 1B,F). Each *n* represented a biological replicate.

Since TNC is a known ligand of TLR4, we analyzed the cortical protein expression. Western blotting for TLR4 revealed no differences in expression between WT and *FMR1* KO groups at P1, P7, P14 or P21 (Figures 2B–F).

Since TLR4 activation by TNC results in the synthesis of IL-6, we analyzed IL-6 cortical protein expression. These experiments revealed a difference in IL-6 protein expression between the WT and *FMR1* KO groups at P7, P14 and P21. At P7, the *FMR1* KO group showed higher IL-6 levels than the WT group (159.2 ± 24.01; *P* < 0.05; *n* = 8/group; Figures 3B,D). At P14, the *FMR1* KO group once again showed different IL-6 levels between the groups (116.4 ± 5.091, *P* < 0.05; *n* = 8/group; Figures 3B,E). There were also differences of IL-6 expression at P21 between the WT and *FMR1* KO groups (145.0 ± 11.46, *P* < 0.001; *n* = 8/group; Figures 3B,F).

### Cell Associated and Secreted Tenascin C and IL-6 Levels Are Altered in *FMR1* KO Primary Astrocyte Cultures

**Figure 4:**
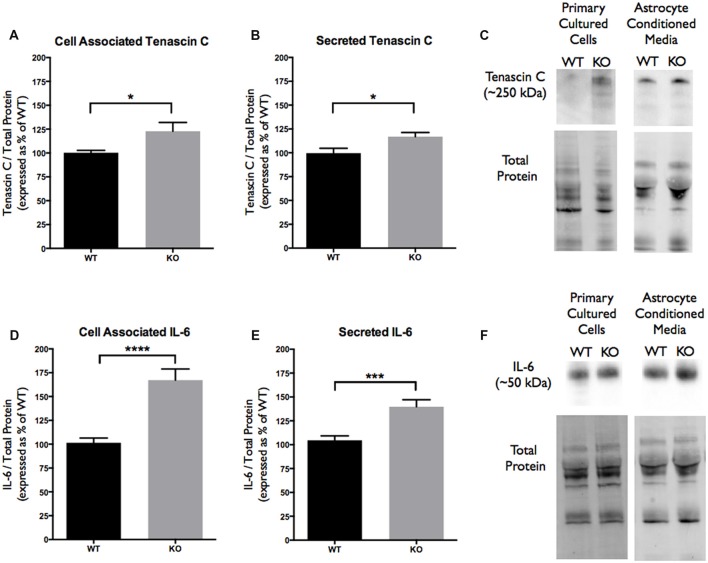
Cell associated and secreted TNC and IL-6 expression is significantly enhanced *in vitro* by *FMR1* KO astrocyte conditioned media (ACM). **(A)** Cell associated expression of TNC in WT (black; *n* = 6) and *FMR1* KO (gray; *n* = 6) primary astrocyte cultured cells grown for 7 days *in vitro*. Representative bands were normalized to the total protein and expressed as a percentage of the average TNC in the WT group. Statistical differences denoted with a single asterisk, *P* < 0.05. **(B)** Secreted expression of TNC in WT (black; *n* = 6) and *FMR1* KO (gray; *n* = 6) ACM. Representative bands were normalized to the total protein and expressed as a percentage of the average TNC in the WT group. Statistical differences denoted with a single asterisk, *P* < 0.05. **(C)** Western blots showing TNC expression (~250 kDa) in primary astrocyte cultured cells and ACM. Corresponding total protein was used as a loading control. **(D)** Cell associated expression of IL-6 in WT (black; *n* = 6) and *FMR1* KO (gray; *n* = 6) primary astrocyte cultured cells grown for 7 days *in vitro*. Representative bands were normalized to the total protein and expressed as a percentage of the average IL-6 in the WT group. Statistical differences denoted with four asterisks, *P* < 0.0001. **(E)** Secreted expression of IL-6 in WT (black; *n* = 6) and *FMR1* KO (gray; *n* = 6) ACM. Representative bands were normalized to the total protein and expressed as a percentage of the average IL-6 in the WT group. Statistical differences denoted with a triple asterisk, *P* < 0.001. **(F)** Western blots showing IL-6 expression (~50 kDa) in primary astrocyte cultured cells and ACM. Corresponding total protein was used as a loading control.

Since TNC is an astrocyte secreted factor, the protein levels in ACM were analyzed and compared between the WT and *FMR1* KO groups. Additionally, cell associated protein levels were also tested to determine if there were any differences between the genotypes. Western blotting experiments revealed an increase of cell associated TNC expression in *FMR1* KO astrocytes (122.8 ± 9.231, *P* < 0.05; *n* = 6/group; Figures 4A,C). Additionally, an increase of secreted TNC was also noted by *FMR1* KO astrocytes (117.0 ± 4.356, *P* < 0.05; *n* = 6/group; Figures 4B,C). Each *n* represented a biological replicate.

As mentioned previously, TNC is a known ligand of TLR4, meaning that activation of TLR4 by TNC may be capable of inducing IL-6 secretion. With differences seen in cell associated and secreted TNC, cell associated and secreted IL-6 protein levels were analyzed. Western blotting experiments revealed changes in cell associated IL-6 expression between the WT and *FMR1* KO astrocytes (167.3 ± 11.61, *P* < 0.0001; *n* = 6/group; Figures 4D,F). Additionally, secreted IL-6 protein levels were also increased by *FMR1* KO astrocytes (139.8 ± 7.296, *P* < 0.001; *n* = 6/group; Figures 4E,F). Each *n* represented a biological replicate.

### Stimulation of TLR4 by Exogenous Tenascin C, LPS and LPS-RS Dysregulated the Secretion of IL-6 by WT and *FMR1* KO Astrocytes

**Figure 5:**
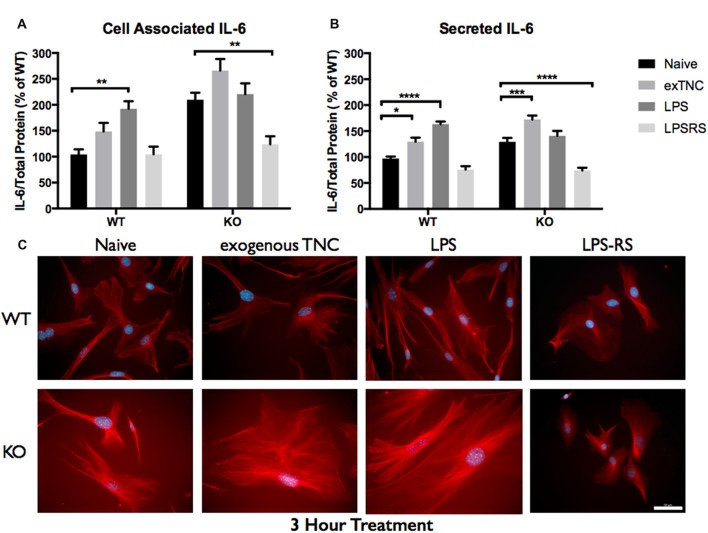
Secreted IL-6 expression post a 3-h treatment is significantly altered following TLR4 activation. **(A)** Cell associated astrocyte expression of IL-6 in WT (*n* = 6/group) and *FMR1* KO (*n* = 6/group) astrocyte cell cultures grown for 7 days *in vitro*. The culture was treated with lipopolysaccharide (LPS; 10 μg/mL), exogenous TNC (10 μg/mL) and LPS-RS (10 μg/mL) for 3 h prior to collection. Representative bands were normalized to the total protein and expressed as a percentage of the average IL-6 in the WT group. Statistical differences denoted with a double asterisk, *P* < 0.01. **(B)** Extracellular astrocyte expression of secreted IL-6 in WT (*n* = 6/group) and *FMR1* KO (*n* = 6/group) astrocyte cell cultures grown for 7 days *in vitro*. The culture was treated with LPS (10 μg/mL), exogenous TNC (10 μg/mL) and LPS-RS (10 μg/mL) for 3 h prior to ACM concentration and collection. Representative bands were normalized to the total protein and expressed as a percentage of the average IL-6 in the WT group. Statistical differences denoted with a single asterisk, *P* < 0.05, with a triple asterisk, *P* < 0.001 and with four asterisks, *P* < 0.0001. **(C)** Cultured cortical astrocytes co-labeled with DAPI (blue) and anti-IL-6 (red) after 3 days *in vitro*, following a 3 h treatment. Images were obtained using a 40x objective with a Zeiss Axioimager M2. Scale bars = 50 μm.

**Figure 6:**
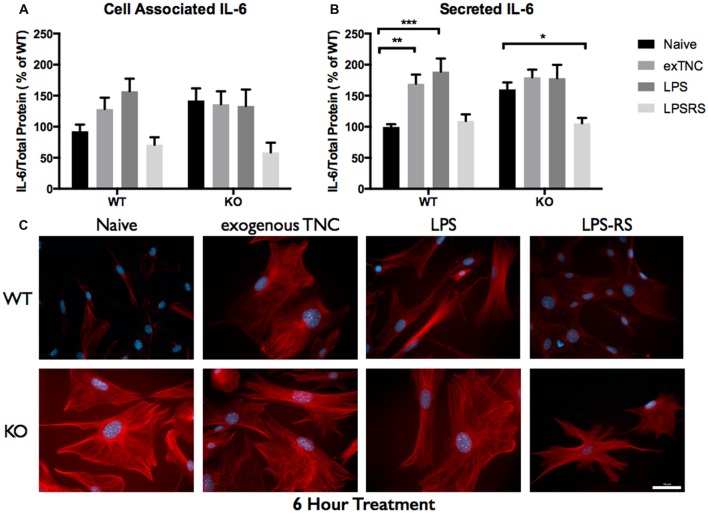
Secreted IL-6 expression post a 6-h treatment is significantly altered following TLR4 activation. **(A)** Cell associated astrocyte expression of IL-6 in WT (*n* = 6/group) and *FMR1* KO (*n* = 6/group) astrocyte cell cultures grown for 7 days *in vitro*. The culture was treated with LPS (10 μg/mL), exogenous TNC (10 μg/mL) and LPS-RS (10 μg/mL) for 6 h prior to collection. Representative bands were normalized to the total protein and expressed as a percentage of the average IL-6 in the WT group. **(B)** Extracellular astrocyte expression of secreted IL-6 in WT (*n* = 6/group) and *FMR1* KO (*n* = 6/group) astrocyte cell cultures grown for 7 days *in vitro*. The culture was treated with LPS (10 μg/mL), exogenous TNC (10 μg/mL) and LPS-RS (10 μg/mL) for 6 h prior to ACM concentration and collection. Representative bands were normalized to the total protein and expressed as a percentage of the average IL-6 in the WT group. Statistical differences denoted with a single asterisk, *P* < 0.05, a double asterisk, *P* < 0.01 and with triple asterisk, *P* < 0.001. **(C)** Cultured cortical astrocytes co-labeled with DAPI (blue) and anti-IL-6 (red) after 3 days *in vitro*, following a 6 h treatment. Images were obtained using a 40× objective with a Zeiss Axioimager M2. Scale bars = 50 μm.

**Figure 7:**
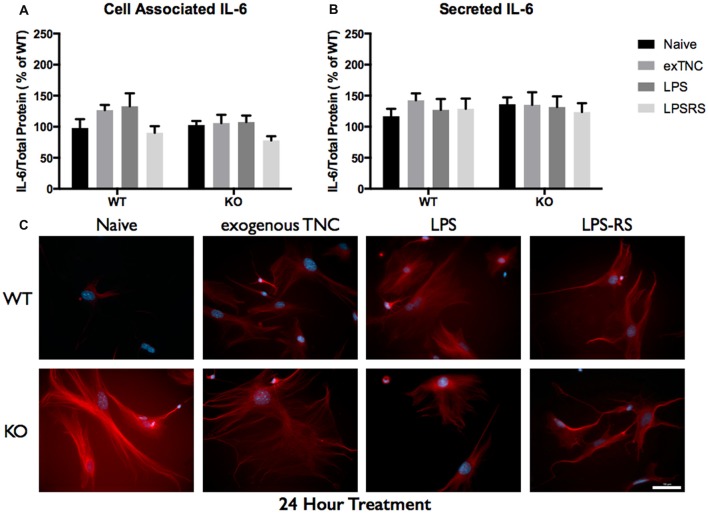
Secreted IL-6 expression post a 24-h treatment is not significantly altered following TLR4 activation. **(A)** Cell associated astrocyte expression of IL-6 in WT (*n* = 6/group) and *FMR1* KO (*n* = 6/group) astrocyte cell cultures grown for 7 days *in vitro*. The culture was treated with LPS (10 μg/mL), exogenous TNC (10 μg/mL) and LPS-RS (10 μg/mL) for 24 h prior to collection. Representative bands were normalized to the total protein and expressed as a percentage of the average IL-6 in the WT group. **(B)** Extracellular astrocyte expression of secreted IL-6 in WT (*n* = 6/group) and *FMR1* KO (*n* = 6/group) astrocyte cell cultures grown for 7 days *in vitro*. The culture was treated with LPS (10 μg/mL), exogenous TNC (10 μg/mL) and LPS-RS (10 μg/mL) for 24 h prior to ACM concentration and collection. Representative bands were normalized to the total protein and expressed as a percentage of the average IL-6 in the WT group. **(C)** Cultured cortical astrocytes co-labeled with DAPI (blue) and anti-IL-6 (red) after 3 days *in vitro*, following a 24 h treatment. Images were obtained using a 40× objective with a Zeiss Axioimager M2. Scale bars = 50 μm.

Since elevated protein levels of TNC and IL-6 were seen in the ACM from the *FMR1* KO mice, it was essential to determine if this differential expression was due to the activation of TLR4. LPS is a known agonist of TLR4, which is capable of inducing multiple pro-inflammatory molecules; one of those being IL-6. To determine if exogenous TNC was capable of inducing IL-6, we treated WT and *FMR1* KO astrocytes with LPS (10 μg/mL) and exogenous TNC (10 μg/mL) for 3, 6 and 24 h (Figures 5–7; Supplementary Figure S1). After the indicated time frames, cells and ACM were collected to determine the cell associated and secreted IL-6 levels by a Western blotting.

When WT astrocytes were treated with LPS for 3 h there was an increase of cell associated IL-6 (*P* < 0.01; *n* = 6/group; Figure 5A) and secreted IL-6 (*P* < 0.0001; *n* = 6/group; Figure 5B). Additionally, when WT and *FMR1* KO astrocytes were treated with exogenous TNC for 3 h, an increase of IL-6 secretion occurred (*P* < 0.05; *n* = 6/group; Figure 5B), however there were no changes in cell associated IL-6 (Figure 5A). Interestingly, when treated with LPS and exogenous TNC for 6 h, an increase of IL-6 secretion was noted only in the WT astrocytes when compared to the Naive group (*P* < 0.001; *n* = 6/group; Figure 6B). By 24 h, there were no changes seen in neither cell associated nor secreted IL-6 protein expression (Figure 7).

LPS-RS is a known antagonist of TLR4, which is capable attenuating pro-inflammatory responses. WT and *FMR1* KO astrocytes were treated with LPS-RS (10 μg/mL) for 3, 6 and 24 h (Figures 5–7; Supplementary Figure S1). After the indicated time frames, cells and ACM were collected to determine cell associated and secreted IL-6 levels by a Western Blot. When FMR1 KO astrocytes were treated with LPS-RS for 3 h, there was a decrease of cell associated IL-6 (*P* < 0.01; *n* = 6/group; Figure 5A), when compared to the Naive group. After 3 h treatments with LPS-RS, ACM from WT astrocytes resulted in no difference in IL-6 secretion, however there was less secreted IL-6 from *FMR1* KO astrocytes (*P* < 0.0001, *n* = 6/group; Figure 5B) when compared to the Naive group. When astrocytes were treated for 6 h with LPS-RS, a difference in IL-6 secretion was only noted in the *FMR1* KO astrocytes (*P* < 0.05, *n* = 6/group; Figure 6B). By 24 h, there was no significant change seen in neither cell associated nor secreted IL-6 protein expression, when the astrocytes were stimulated with LPS-RS (Figure 7).

### *FMR1* KO Astrocyte Conditioned Media Increases Co-localized Excitatory Synaptic Puncta

**Figure 8:**
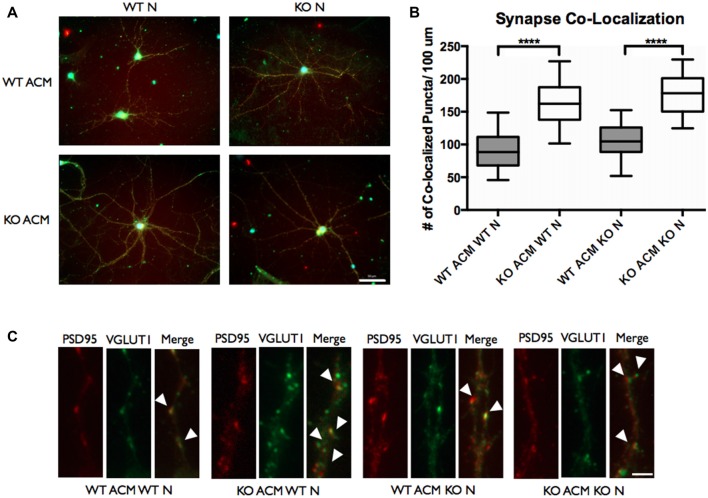
The number of vesicular glutamate transporter 1 (VGLUT1)/post synaptic density protein 95 (PSD95) co-localized puncta of WT and *FMR1* KO neurons was increased when plated with ACM from *FMR1* KO astrocytes, compared to those plated with media from WT astrocytes. **(A)** WT and *FMR1* KO neurons with ACM from WT and *FMR1* KO astrocytes maintained for 12 days *in vitro*, co-labeled with antibodies against VGLUT1 and PSD95. Scale bars = 50 μm. **(B)** Measures of intracortical synapse number (identified by the co-localization of VGLUT1 and PSD95 puncta) obtained from cultures containing WT or *FMR1* KO neurons, supplemented with ACM from WT or *FMR1* KO astrocytes (*n* = 4; 40 neurons /condition). Statistical differences denoted with four asterisks, *P* < 0.0001. **(C)** WT and *FMR1* KO neurons supplemented with ACM from WT or *FMR1* KO astrocytes and maintained for 12 days *in vitro*. White arrows indicate co-localized PSD95 (red) and VGLUT1 (green) puncta. Images were obtained using a 40× objective with a Zeiss Axioimager M2. Scale bars = 10 μm.

Autism spectrum disorders have been speculated to arise from functional changes in neuronal circuitry, and they have been associated with an imbalance in excitatory/inhibitory synaptic transmission. To investigate whether the elevated IL-6 in *FMR1* KO astrocytes could impact synapse formation we used antibodies against synaptic vesicle proteins. With our previous finding of increased cell associated and secreted IL-6, we used ACM from WT and *FMR1* KO astrocytes to determine the number of excitatory synaptic puncta. Antibodies against VGLUT1 and PSD95 were used to analyze the co-localization of the pre- and post-synaptic markers (Figure 8). We examined a dramatic increase of co-localized excitatory puncta when WT and *FMR1* KO neurons were supplemented with *FMR1* KO ACM and maintained for 12 days *in vitro* (*P* < 0.0001, *n* = 4, 40 neurons per condition, Figure 8).

## Discussion

The first few weeks of postnatal development consist of highly regulated events involving synapse growth, formation and pruning (Faissner et al., 2010; Gatto and Broadie, 2010). This complex and dynamic process requires a coordinated exchange of signals from neurons and glia (Cheng et al., 2012). In many disorders of the nervous system, the structure and function of synapse formation is strained, and thereby translated to cognitive and behavioral deficits (Cheng et al., 2012; Contractor et al., 2015). Recently, astrocytes have emerged as an essential cell type for the regulation of synaptic connectivity. Given their close proximity to neurons, astrocytes are able to regulate synapse formation through secreted and contact mediated factors (Faissner et al., 2010; Jones and Bouvier, 2014). A multitude of soluble factors secreted by astrocytes are responsible for synaptogenesis and synaptic function. Previous research from our lab has demonstrated that Hevin, SPARC and Thrombospondin-1 are soluble factors that can rescue afferent spine morphology in the *FMR1* KO mouse model (Jacobs and Doering, 2010; Cheng et al., 2016; Wallingford et al., 2017). Thus, the discovery of other astrocyte secreted factors is essential to understand the extracellular impact of the environment on neurons.

### TNC, TLR4 and IL-6 Expression in the Cortex and Primary Astrocyte Cultures in WT and *FMR1* KO Astrocytes

In this study, we examined the interaction and activation of TLR4 by the astrocyte secreted factor, TNC. Such activation would result in the synthesis of pro-inflammatory cytokines such as IL-6, which is ultimately known to be involved in the formation of excitatory synapses (Tancredi et al., 2000; Wei et al., 2011). This study is the first to determine the relationship between TNC and IL-6, through the activation of TLR4 in astrocytes and in FXS. Interestingly, we discovered an up-regulation of TNC and IL-6 in the *FMR1* KO cortices at P14 and P21 (Figures 1E,F, 3E,F). TNC expression was found to be down-regulated at P7 (Figure 1D), potentially due to upstream factors such as intracellular signals that are controlled by soluble factors, integrins and other mechanical forces (Jones and Jones, 2000). Another mechanism for the elevated TNC expression could be due to astrocyte reactivity. First evidence of astrocyte reactivity in the FXS mouse model has been shown by the Pacey lab, making this a viable explanation as to why there is an increase of TNC in the *FMR1* KO model (Pacey et al., 2015). Additionally, developmental expression of TNC was found to be down-regulated in WT astrocytes, however this does not seem to be the case for TNC expression by *FMR1* KO astrocytes. Thus, these results do complement previous findings that TNC is developmentally down-regulated in a normal functioning system (Joester and Faissner, 1999). Interestingly, IL-6 expression still remained elevated at P7, even though TNC expression was found to be down-regulated (Figures 1, 3). This could be explained by IL-6 regulation through the JAK/STAT3 pathway, as IL-6 has the potential to recruit JAK and phosphorylate STAT3, thus increasing the production and secretion of IL-6 (Beurel et al., 2009; Samavati et al., 2009; Greenhill et al., 2011). However, since we bred WT and *FMR1* KO mice from separate colonies, we cannot exclude the possibility that the effects we observe are influenced by differences in maternal care.

In rodents, the critical time-span for synaptogenesis occurs in the first 2 weeks of life, while maturation of the synapses occurs approximately between P14 and P21 (Gatto and Broadie, 2010; Cheng et al., 2012). The elevation of TNC protein levels at these critical time points may suggest that there is more involvement of TNC in synaptogenesis than previously investigated. The maintenance of plasticity in the brain correlates with the expression of extracellular glycoproteins such as TNC, with recent studies suggesting that TNC may be involved in synapse function through the modulation of intracellular Ca^2+^ levels (Dityatev and Schachner, 2003; Faissner et al., 2010; Barros et al., 2011).

TNC is expressed at low amounts in healthy tissue, although up-regulation is seen in tissue injury and during states of inflammation (Udalova et al., 2011; Stamenkovic et al., 2016). This has been shown to trigger TLR4 activation in macrophages, dendritic cells, neutrophils and synovial fibroblasts (Midwood et al., 2009; Wei et al., 2011). In the CNS, there is a vast amount of TLR4 expression by neurons, microglia, as well as astrocytes (Gorina et al., 2011; Shen et al., 2016). This study determined that the expression levels of cortical TLR4 are similar in both the WT and *FMR1* KO mouse model (Figure 2). A recent study suggested that astrocytic activation of TLR4 through the Myd88 mediated pathway by LPS, induces pro-inflammatory cytokines and promotes excitatory synaptogenesis in the second postnatal week in mice (Shen et al., 2016). This ultimately prompted the question whether TNC is capable of activating TLR4 in astrocytes to promote excitatory synaptogenesis through cytokine synthesis.

The pro-inflammatory cytokine of interest in this study was IL-6, as it has been implicated in ASD and in the formation of excitatory synapses (Wei et al., 2011, 2012). Although primary functions of IL-6 have been linked to neuroimmune responses that seem to be involved in physiological brain development, a recent study has suggested that IL-6 is increased in the cerebellum of autistic patients, which in turn alters synapse formation (Wei et al., 2011). Additionally, primary hippocampal neuron cultures, treated with exogenous IL-6 have shown to significantly reduce Group II-metabotropic receptors (mGluR2/3) and L-type Ca^2+^ channels (Vereyken et al., 2007). This is of interest since in the valproate-induced model of autism, mGluR2/3 protein and mGluR2 mRNA has been shown to be reduced (Jacobs and Doering, 2010). Major changes in Ca^2+^ signaling have also been noted due to the absence of FMRP, which may result from the reduced mRNA for the L-type Ca^2+^ channels (Contractor et al., 2015).

### WT and *FMR1* KO Astrocyte Response to Immunological Challenges

The TLR receptor family has been mediating host defense infection and injury by recognizing pathogen and damage associated molecular patterns (PAMPS and DAMPS; Trotta et al., 2014). Upon activation with bacterial endotoxin LPS, TLRs promote responses such as induction of cytokines mediated by protein tyrosine kinases, mitogen-activated protein kinases (MAPKs) and transcription factors such as nuclear factor κB (NFκB; Gorina et al., 2011). To determine if *FMR1* KO astrocytes respond differently to an immunological challenge, we treated WT and *FMR1* KO astrocytes with LPS and examined the cultures with Western blots 3, 6 and 24 h post-treatment (Figures 5–7). Only the WT astrocytes responded to LPS stimulation after 3 and 6 h treatments, as seen by an increase of secreted IL-6 in contrast to the *FMR1* KO astrocytes, where secreted levels remained comparable to the Naive group. This complements a previous report which observed no alterations in cultured primary microglia lacking FMRP when treated for 6 h with LPS (100 ng/mL; Yuskaitis et al., 2011). To further analyze how TLR4 is capable of IL-6 induction through endogenous stimuli such as TNC, we treated WT and *FMR1* KO astrocytes with TNC for 3, 6 and 24 h, and analyzed IL-6 protein expression with Western blotting (Figures 5–7). We have shown here that TNC is capable of activating TLR4 in the astrocyte and promoting cytokine secretion in WT astrocytes. In particular, the FBG domain has been identified as the domain to cause spontaneous release of IL-6 via the MyD88 dependent pathway (Midwood et al., 2009). *FMR1* KO astrocytes seemed to respond to exogenous TNC treatment only when treated for 3 h (Figure 5B). Since the *FMR1* KO astrocytes already have increased cell associated and secreted TNC levels, the system may have become oversaturated thus being unable to respond to the agonist when treated for longer periods of time (Figures 6B, 7B). To determine whether *FMR1* KO astrocytes respond differently to TLR4 antagonist treatments, both WT and *FMR1* KO astrocytes were treated with LPS-RS for 3, 6 and 24 h. Interestingly, only *FMR1* KO astrocytes had the ability to decrease the amount of secreted IL-6 when the TLR4 receptor was antagonized for 3 and 6 h (Figures 5B, 6B). Antagonist treatment effects were not seen in WT astrocytes potentially because *FMR1* KO astrocytes were more sensitive to the LPS-RS treatment. By 24 h, both the WT and *FMR1* KO may have become desensitized as no treatment effect is seen for either the TLR4 agonist and antagonist treatments (Figure 7).

### Increase of VGLUT1/PSD95 Synapse Co-localization of WT and *FMR1* KO Neurons Supplemented With Conditioned Media From *FMR1* KO Astrocytes

Synapses are points of cellular communication, which mediate neuronal connections. They rely on proper formation, maintenance and function, that are dependent by the number, type and connectivity patterns of neuronal circuitries (Chung et al., 2015). Improper synapse number, which ultimately leads to functional dysregulation, may cause neurodevelopmental disorders such as FXS. Keeping in mind the potential role that IL-6 may play in synaptic development (Wei et al., 2011), we sought to determine whether WT and *FMR1* KO neurons developed a different number of synaptic connections when cultured with ACM from *FMR1* KO astrocytes, compared to those cultured with ACM from WT astrocytes. We co-labeled these neurons with antibodies against VGLUT1 and PSD95. Intracortical synaptic connections were determined by co-localization of the pre- and post- synaptic markers. Interestingly we found an increased number of excitatory synaptic connections when WT and *FMR1* KO neurons were supplemented with ACM from *FMR1* KO astrocytes (Figure 8). It is worth noting that ACM from WT astrocytes did not impact synaptic formation (Figure 8).

It is well established that abnormal spine morphology and density are the major players in FXS research, however, only a handful of studies directly quantify the changes in excitatory synapses (Pfeiffer and Huber, 2009; Contractor et al., 2015; Yang et al., 2016). To study the changes of excitatory synaptic density in more detail, we focused on the co-localization of VGLUT1 and PSD95. VGLUT1 is expressed in the majority of cortical-cortical synapses, in particular, these excitatory synaptic connections have been found to be elevated in layer IV and layer V of the mouse somatosensory cortex in FXS (Wang et al., 2014). Since FXS is associated with hypersensitivity to sensory stimuli (Gatto and Broadie, 2010), an altered cortical-cortical connectivity could explain the deficits of processing sensory information.

The complexity of neuronal connections is dependent on the modulation of cell adhesion molecules (CAMs), which regulate synapse formation, maturation and plasticity (McGeachie et al., 2011; Lee et al., 2013). Recently, IL-6 over-expression in granule cells was found to affect cell adhesion and migration, which suggests that IL-6 could be implicated in the function of CAMs (Wei et al., 2011). In our study we have found that cell associated and secreted IL-6 is elevated in *FMR1* KO astrocytes, which further supports the hypothesis that IL-6 has an impact on CAMs. Additionally, TNC is also known to interact directly with CAMs (Zacharias et al., 1999; Rigato et al., 2002), supporting the argument that both TNC and IL-6 are implicated in synaptic development.

Here, we not only show that elevated levels of endogenous TNC is present in the *FMR1* KO astrocytes, but also that exogenous TNC is capable of inducing secretion of IL-6. With already elevated secreted levels of IL-6 present in astrocytes lacking FMRP, it was crucial to determine if this was the doing of TNC. Understanding the mechanism by which endogenous activators of TLR4 such as TNC mediate the secretion of IL-6 and other cytokines may help determine which astrocyte secreted factors and to what extent these factors impact synaptic dysregulation. Our findings thus show that an imbalance of astrocyte secreted factors could result in an imbalance of neuronal circuitries in FXS. Further experiments involving the NF-κB pathway, the JAK/STAT3 signaling pathway and integrin contribution could further provide evidence to support the hypothesis of IL-6 being one of these important astrocyte secreted factors that partake in synapse development.

## Conclusion

In conclusion, our study demonstrates that TNC and IL-6 are both significantly increased in the *FMR1* KO cortex and *FMR1* KO astrocytes at various stages of cortical development. We also showed that TNC has the capability of inducing the secretion of IL-6 in WT and *FMR1* KO astrocytes *in vitro*. This study also investigated how *FMR1* KO astrocytes react to TLR4 agonists and antagonist in comparison to WT astrocytes. We speculate that the *FMR1* KO system is oversaturated by TNC, thus being unable to affect IL-6 secretion when treated with additional TLR4 agonists for long periods of time. We also speculate that *FMR1* KO astrocytes are more sensitive to some treatments such as LPS-RS. Ultimately, we can confirm the importance of astrocyte secreted factors in synaptic formation and development, however additional experiments are required to determine to what extent this process is driven by IL-6. These findings suggest that elevated IL-6, which has been previously noted in the autistic brain, could be the contributing factor in abnormal synapse formation, as shown by an increase of excitatory synapses in FXS, which would ultimately contribute to the development of autism.

## Author Contributions

VK: conception and design, collection and/or assembly of data, data analysis and interpretation, manuscript writing, final approval of manuscript. LD: conception and design, financial support, provision of study material, final approval of manuscript.

## Conflict of Interest Statement

The authors declare that the research was conducted in the absence of any commercial or financial relationships that could be construed as a potential conflict of interest.

## Supplementary Material

FIGURE S1 Cellular localization of IL-6 in WT and *FMR1* KO astrocytes treated with exogenous TNC, LPS and LPSRS. Extracellular astrocyte expression of secreted IL-6 in WT (*n* = 6/group) and *FMR1* KO (*n* = 6/group) astrocyte cell cultures grown for 7 days *in vitro*. The culture was treated with LPS (10 μg/mL), exogenous TNC (10 μg/mL) and LPS-RS (10 μg/mL) prior to ACM concentration and collection. Cultured cortical astrocytes co-labeled with 4’,6-diamidino-2-Phenylindole (DAPI; blue) and anti-IL-6 (red) after 3 days *in vitro*, following treatment. Images were obtained using a 40× objective with a Zeiss Axioimager M2. Scale bars = 10 μm.
