# Supplementary figures and images for: Regulation of IL-6 Secretion by Astrocytes via TLR4 in the Fragile X Mouse Model
Source: Front Mol Neurosci. 2018 Aug 3;11:272. doi: 10.3389/fnmol.2018.00272 (PMC6085486; doi:10.3389/fnmol.2018.00272)

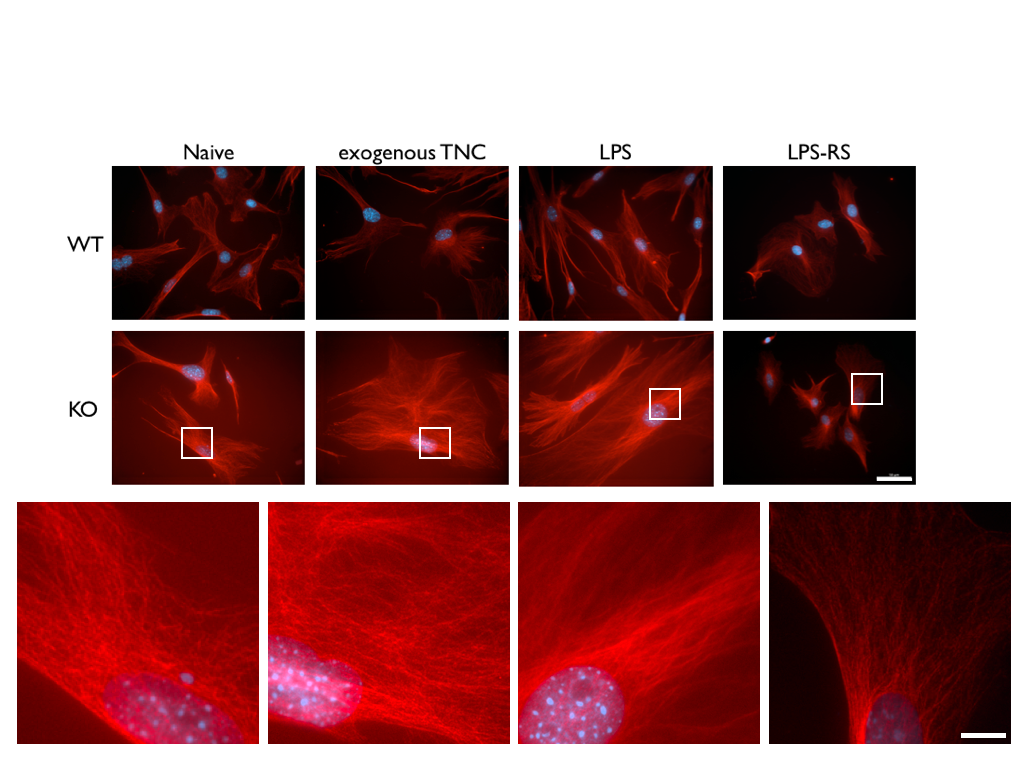

Supplement: FIGURE S1 — Cellular localization of IL-6 in WT and FMR1 KO astrocytes treated with exogenous TNC, LPS and LPSRS. Extracellular astrocyte expression of secreted IL-6 in WT (n = 6/group) and FMR1 KO (n = 6/group) astrocyte cell cultures grown for 7 days in vitro. The culture was treated with LPS (10 μg/mL), exogenous TNC (10 μg/mL) and LPS-RS (10 μg/mL) prior to ACM concentration and collection. Cultured cortical astrocytes co-labeled with 4’,6-diamidino-2-Phenylindole (DAPI; blue) and anti-IL-6 (red) after 3 days in vitro, following treatment. Images were obtained using a 40× objective with a Zeiss Axioimager M2. Scale bars = 10 μm. [file Image_1.tiff]
